# The transmembrane tyrosines Y56, Y91 and Y167 play important roles in determining the affinity and transport rate of the rabbit proton-coupled peptide transporter PepT1

**DOI:** 10.1016/j.biocel.2009.04.014

**Published:** 2009-11

**Authors:** Myrtani Pieri, Christine Gan, Patrick Bailey, David Meredith

**Affiliations:** a School of Life Sciences, Oxford Brookes University, Headington, Oxford OX3 OBP, UK; b Department of Physiology, Anatomy and Genetics, University of Oxford, Le Gros Clark Building, South Parks Road, Oxford OX1 3QX, UK; c The Dean's Office, Faculty of Natural Sciences, Keele University, Keele, Staffordshire ST5 5BG, UK

**Keywords:** Epithelia, Membrane transport, SLC15a1, Nutrient absorption, Protein structure–function, Site-directed mutagenesis

## Abstract

The mammalian proton-coupled peptide transporter PepT1 is widely accepted as the major route of uptake for dietary nitrogen, as well as being responsible for the oral absorption of a number of classes of drugs, including β-lactam antibiotics and angiotensin-converting enzyme (ACE) inhibitors. Using site-directed mutagenesis and zero-*trans* transport assays, we investigated the role of conserved tyrosines in the transmembrane domains (TMDs) of rabbit PepT1 as predicted by hydropathy plots.

All the individual TMD tyrosines were substituted with phenylalanine and shown to retain the ability to traffic to the plasma membrane of *Xenopus laevis* oocytes. These single substitutions of TMD tyrosines by phenylalanine residues did not affect the proton dependence of peptide uptake, with all retaining wild-type PepT1-like pH dependence. Individual mutations of four of the nine TMD residue tyrosines (Y64, Y287, Y345 and Y587) were without measurable effect on PepT1 function, whereas the other five (Y12, Y56, Y91, Y167 and Y345) were shown to result in altered transport function compared to the wild-type PepT1.

Intriguingly, the affinity of Y56F-PepT1 was found to be dramatically increased (approximately 100-fold) in comparison to that of the wild-type rabbit PepT1. Y91 mutations also affected the substrate affinity of the transporter, which increased in line with the hydrophilicity of the substituted amino acid (F > Y > Q > R). Y167 was demonstrated to play a pivotal role in rabbit PepT1 function since Y167F, Y167R and Y167Q demonstrated very little transport function. These results are discussed with regard to a proposed mechanism for PepT1 substrate binding.

## 1 Introduction

The proton-coupled di- and tri-peptide transporter PepT1 (SLC15a1) is the major route of uptake of dietary nitrogen from the intestine, and is also important along with the higher affinity gene product PepT2 (SLC15a2) in the re-absorption of filtered peptides in the kidney (Daniel and Kottra, 2004; Meredith and Boyd, 2000). In addition, PepT1 is the route of entry of a wide class of orally bio-available pharmaceutically important compounds, including the β-lactam antibiotics, angiotensin-converting enzyme (ACE) inhibitors, antiviral and anticancer agents (Terada and Inui, 2004). Although these therapeutic compounds are not di- or tri-peptides, they are carried by virtue of their similar 3D shape to endogenous substrates, i.e. they are peptidomimetic, and modelling of the substrate binding site from the features in common of this huge and diverse range of substrates has led to predictions concerning which parts of the PepT1 protein may be important. For example, a substrate template model has been developed by several groups (Bailey et al., 2000, 2006; Biegel et al., 2005) which allows prediction of binding affinity for a potential substrate.

Rabbit PepT1 (rPepT1) is a 707 amino acid protein, with twelve transmembrane spanning domains (TMDs) as predicted by hydropathy plots (Fei et al., 1994) and confirmed experimentally in human PepT1 (hPepT1) by epitope mapping (Covitz et al., 1998). In the absence of a crystal structure, attempts have been made to computer model the PepT1 transporter itself (Bolger et al., 1998; Meredith and Price, 2006; Pedretti et al., 2008), with site-directed mutagenesis (SDM) used to test hypotheses generated by these models. From computer modelling, it has previously been proposed that certain TMD tyrosines in the hPepT1 transporter may contribute to its transport function (Chen et al., 2000; Lee et al., 1999). Additionally, it has been shown that selective chemical modification of tyrosine residues in rat brush border membrane vesicles results in the inhibition of both peptide and β-lactam antibiotics transport (Kramer et al., 1990). Nevertheless, an investigation of the role of tyrosine residues embedded in the predicted 12TMDs of PepT1 has never taken place in a systematic way until now.

It is known that aromatic residues such as tyrosine, phenylalanine and tryptophan stabilize positive charges within the membrane electric field through the cation–π interaction (Dougherty, 1996). Tyrosine residues have been implicated in the selectivity of potassium channels (Kumpf and Dougherty, 1993) and their susceptibility to inhibition by tetraethylammonium (TEA, Ahern et al., 2006). Similarly, for voltage-gated sodium channels, tyrosines are involved in both the selection of the carried ion and the binding of inhibitors such as calcium (Santarelli et al., 2007a) and tetrodotoxin (Santarelli et al., 2007b), the latter long known to be more potent in its cationic form (Camougis et al., 1967). Cation–π interactions are also important in the binding of substrates to membrane transport proteins, such as in the vesicular acetylcholine transporter (Ojeda et al., 2004), the sodium-bile acid cotransporter (Banerjee et al., 2008), and the dopamine transporter (Kniazeff et al., 2008). Tyrosine residues are also known to have the ability to donate and receive protons due to their unique chemical structure; although this concept of hydrogen atom transfer is best understood in chemistry (reviewed in Mayer, 2004), there are biological correlates (for examples in photosynthesis; Tommos, 2002). The characteristics and known roles of tyrosines make them an appealing candidate for involvement in the substrate translocation pathway of PepT1, as has previously been suggested in the literature for protons (Bolger et al., 1998) or for stabilising the protein during the translocation cycle (Pieri et al., 2008).

In this study, a sequential approach was followed in order to identify the key TMD tyrosines for rPepT1 function. This was accomplished by individually mutating the TMD tyrosines *via* SDM in conjunction with functional expression of the mutant transporters in *Xenopus laevis* oocytes. Residues that had previously been identified by computational modelling to be important in PepT1 function were tested in addition to ones not previously considered essential. Following a basic screening, the more interesting residues were examined in more detail.

Some of these data have been previously published in abstract form (Pieri et al., 2006, 2004).

## 2 Materials and methods

### 2.1 Site-directed mutagenesis of the PepT1 gene

Oligonucleotides were custom synthesised (Sigma-Genosys, UK) for the following sequences for forward primers as shown in Table 1 (residues in bold are changed from wild-type rPepT1). Reverse primers for the PepT1 mutant PCR reactions were the reverse compliment of the forward primers. The site-directed PepT1 mutants were generated using the Quikchange protocol (Stratagene), and the resulting constructs confirmed by DNA sequencing (GeneService, UK).

### 2.2 cRNA synthesis and oocyte injection

rPepT1 constructs were linearised with *XbaI* (New England Biolabs, UK) and cRNA generated by *in vitro* transcription (T7 mMessage mMachine, Ambion, Cambridgeshire, UK). *Xenopus laevis* oocytes were obtained under MS222 anaesthesia (0.2%, w/v) in accordance with the UK Animals (Scientific Procedures) Act, 1986, and maintained at 18 °C in modified Barth's medium (88 mM NaCl, 1 mM KCl, 0.82 mM MgSO_4_, 2.4 mM NaHCO_3_, 0.42 mM CaCl_2_, 10 mM Hepes, 5 mM sodium pyruvate, 50 μg ml^−1^ gentamicin (Fluka, Poole, UK), adjusted to pH 7.6 with 1 M NaOH). Transport measurements were performed at least 72 h after micro-injection of oocytes with 27nl cRNA (1 μg/μl), with medium changed daily.

### 2.3 Transport experiments

Uptake of [^3^H]-d-Phe-l-Gln (17.4 Ci/mmole, custom synthesised, Cambridge Research Biochemicals, Stockton-on-Tees, UK) was performed as previously described (Meredith, 2004). Briefly, 5 oocytes were incubated in 100 μl of uptake medium (95 mM NaCl, 2 mM KCl, 1 mM CaCl_2_, 0.42 mM MgCl_2_, 10 mM Tris/Hepes pH 7.4 or Tris/Mes pH 5.5) with tracer (0.4 μM) [^3^H]-d-Phe-l-Gln. After incubation, the oocytes were washed sequentially five times in 1 ml of ice-cold 120 mM NaCl solution, lysed individually with 100 μl 2% (w/v) SDS and liquid scintillation counted. As a control non-injected oocytes were also incubated in uptake medium with d-Phe-l-Gln as above.

The affinity of wild-type and mutant rPepT1 transporters were assessed by competition studies with 0.4 μM [^3^H]-d-Phe-l-Gln and Gly-l-Gln present in the uptake medium in concentrations from 0 to 2 mM using the protocol above, and the *Ki* calculated using the method of Deves and Boyd (1989).

### 2.4 Membrane expression determination by luminometry

All tyrosine mutants were generated using as template the rPepT1-FLAG construct, which has previously been shown to give a fully functional transporter (Panitsas et al., 2006). Luminometry assays were performed as described previously (Panitsas et al., 2006). Briefly, rPepT1-FLAG- or mutant rPepT1-FLAG-expressing and non-injected control oocytes were incubated in ice-cold filtered Barth's medium + 1% BSA for 30 min and then in 1:500 HRP-conjugated anti-FLAG antibody (Invitrogen, UK, 1:500 in Barth's medium + 1% BSA) for 60 min at 4 °C (5 oocytes/100 μl). After incubation, oocytes were sequentially washed (10 min, 14 times) with Barth's medium + 1% BSA to remove any unbound antibody. After washing, individual oocytes were placed in a vial containing 50 μl of SuperSignal ELISA Femto Maximum Sensitivity Substrate (Pierce/Perbio, UK). Luminometer (Turner-Designs 20/20, Promega) readings for at least 15 oocytes per data point were taken immediately.

### 2.5 Data analysis

All data are expressed as mean ± SEM after subtraction of data from non-injected control oocytes. Significance was tested using paired or unpaired Student's *t*-test where appropriate. The *n* value refers to the number of oocyte preparations that were used, with at least 5 oocytes per data point used in individual experiments.

## 3 Results

### 3.1 Expression of the tyrosine to phenylalanine mutations in *Xenopus* oocytes

**Fig. 1:**
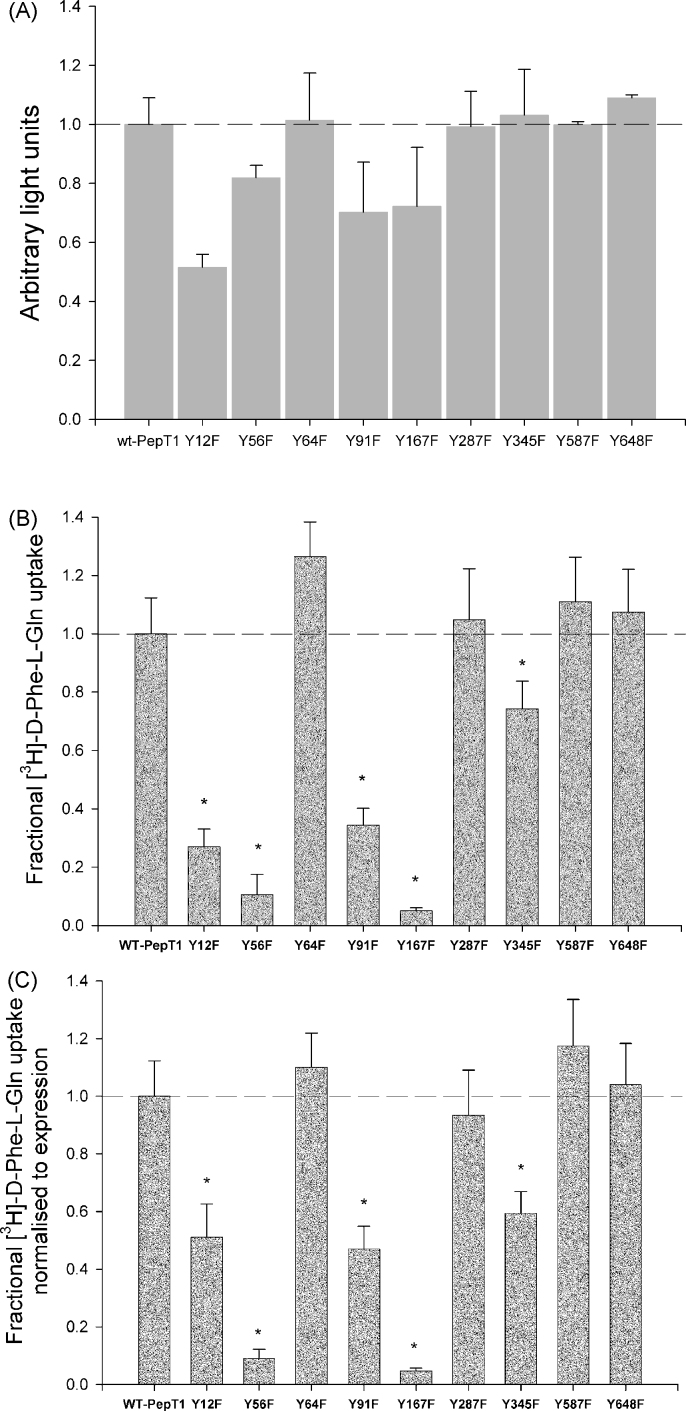
(A) Surface expression quantification of all TMD tyrosine to phenylalanine mutants using luminometry against the FLAG epitope incorporated in each mutant. Wild type rPepT1-FLAG (wt-PepT1) surface expression was used as a positive control, and non-injected oocyte values were subtracted from all data as a negative control. Results are normalised to wild-type and expressed as mean ± SEM, *n* = 3 separate experiments, >20 oocytes per experiment. (B) Uptake of 0.4 μM [^3^H]-d-Phe-l-Gln into *Xenopus* oocytes expressing wild-type rPepT1 (wt-Pept1) or the individual tyrosine mutants. Non-injected control oocyte values have been subtracted from the data. Each data point is expressed as the mean ± SEM, *p < 0.05, *n* ≥ 3 separate experiments. The wild-type rPepT1 uptake corresponds to 342 ± 37 fmol peptide/(oocyte h). (C) Uptake of 0.4 μM [^3^H]-d-Phe-l-Gln into *Xenopus* oocytes expressing wild-type rPepT1 (wt-PepT1) or individual tyrosine mutants corrected for surface expression. Non-injected control oocyte values have been subtracted from the data. Note that adjustment has been made to normalize uptake to the level of protein expression for each mutant in order to express the functional effect of each mutation. Each data point is the mean ± SEM, *p < 0.05, *n* ≥ 3 separate experiments.

Surface expression quantification (as shown in Fig. 1A) of all TMD tyrosine to phenylalanine mutants using luminometry against the FLAG epitope showed that all mutants retained their ability to reach the *Xenopus* oocyte membrane.

### 3.2 Functional characteristics of the tyrosine to phenylalanine mutants using the model neutral dipeptide [^3^H]-d-Phe-l-Gln

As depicted in Fig. 1B, the functional activities of the wild-type and nine individually mutated rPepT1s expressed in *Xenopus* oocytes were evaluated by measuring [^3^H]-d-Phe-l-Gln uptake at extracellular pH 5.5 for 1 h. Data are normalised to the wild-type rate of transport. It was found that the Y56F-rPepT1 and Y167F-rPepT1 mutants significantly decreased the rate of [^3^H]-d-Phe-l-Gln uptake by almost 90% as compared to the wild-type at pHout 5.5 (*p* < 0.01, *n* ≥ 4). Similarly, the Y12F-rPepT1, Y91F-rPepT1 and Y345F-rPepT1 mutants significantly decreased [^3^H]-d-Phe-l-Gln uptake rate at pHout 5.5, but to lesser extent (*p* < 0.01, *n* ≥ 4).

When the uptakes were corrected for surface expression (Fig. 1C), the same pattern of results was observed, i.e. the rate of [^3^H]-d-Phe-l-Gln uptake per transporter molecule for the Y12F-, Y56F-, Y91F-, Y167F- and Y345F-rPepT1 was significantly reduced, whereas that of the Y64F-, Y287F-, Y587F- and Y648F-rPepT1 mutant constructs was not altered from the wild-type transporter.

### 3.3 Is uptake by the tyrosine to phenylalanine mutants pH dependent?

**Fig. 2:**
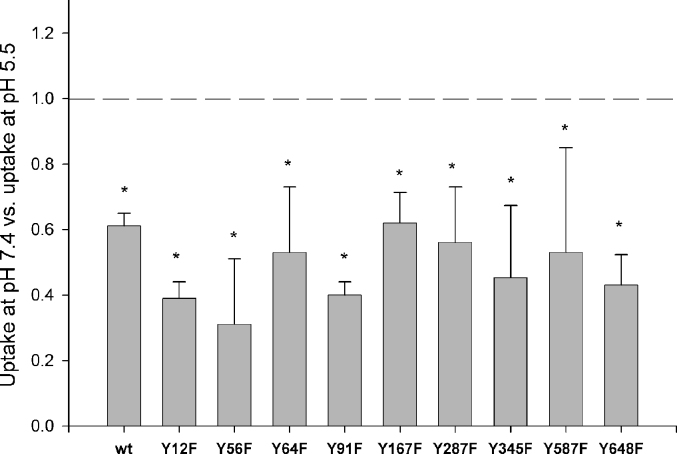
Comparison of pH dependence of [^3^H]-d-Phe-l-Gln uptake by the wild-type rPepT1 (wt) and individual tyrosine mutants. Uptake of [^3^H]-d-Phe-l-Gln by oocytes was measured at extracellular pH 5.5 and pH 7.4 after one hour incubation. Values represent mean ± SEM for three separate oocyte preparations and are normalized to the uptake at pHout 5.5 for each construct, *p < 0.01, *n* = 3, Student's *t*-test compared to pHout 7.4.

As illustrated in Fig. 2, all mutants exhibited a significant decrease in the rate of [^3^H]-d-Phe-l-Gln uptake by alkalization of the extracellular pH from 5.5 to 7.4. Therefore, all tyrosine to phenylalanine mutants, even the ones that show reduced transport function, still follow the wild-type-like pH dependent mode of transport.

### 3.4 Kinetic determination of the tyrosine to phenylalanine mutants that demonstrate decreased transport function

**Fig. 3:**
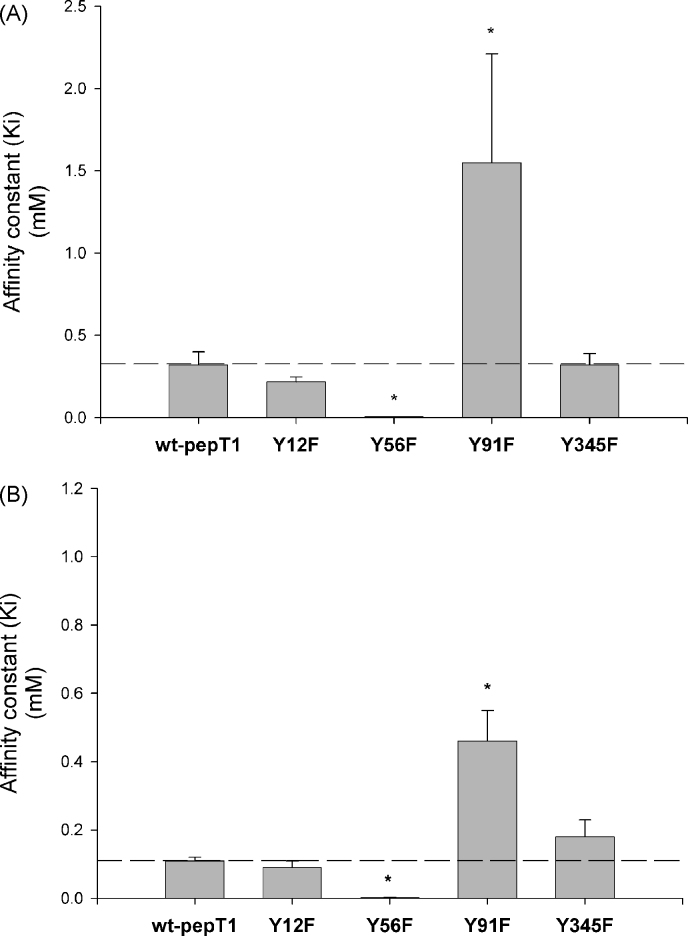
Graphs show the affinity constants (*Ki*, mM) for the Y12F-, Y56F-, Y92F- and Y345F-rPepT1 mutants for both pHout 5.5 (A) and 7.4 (B). The uptake of 0.4 μM [^3^H]-d-Phe-l-Gln into oocytes was measured in the presence of increasing concentrations of the non-radioactive (cold) Gly-l-Gln dipeptide. Each *Ki* was calculated from the best fit of the data to Michaelis–Menten kinetics for binding to a single site (Deves and Boyd, 1989), *p < 0.05, *n* = 3, Student's *t*-test compared to wild-type.

The affinity constants (*Ki*) were determined using the method described by Deves and Boyd (1989). Comparing the *Ki* constants for Gly-l-Gln at pH 5.5 (Fig. 3A) revealed that the binding affinity for the Y12F and the Y345F mutant transporters (0.22 ± 0.02 mM and 0.32 ± 0.07 mM, respectively) were not significantly altered from that of the wild-type protein (0.32 ± 0.08 mM, *p* > 0.05, *n* = 3). The same was observed when pHout 7.4 (Fig. 3B) where the *Ki* constants for the Y12F and the Y345F mutant transporters were 0.09 ± 0.02 mM and 0.18 ± 0.05 mM, respectively, as compared with the wild-type *Ki* of 0.11 ± 0.01 mM (*p* > 0.05, *n* = 3). In comparison, the Y91F-rPepT1 transporter exhibited significantly reduced affinity as compared to the wild-type protein at both pH 5.5 and 7.4 (1.40 ± 0.31 mM versus 0.33 ± 0.11 mM at pHout 5.5 and 0.46 ± 0.09 mM versus 0.11 ± 0.01 mM at pHout 7.4, respectively, *p* < 0.05, *n* = 3). Interestingly, the Y56F-rPepT1 mutant showed a dramatically increased binding affinity for d-Phe-l-Gln as compared to the wild-type protein (0.006 ± 0.001 mM versus 0.32 ± 0.08 mM at pHout 5.5 and 0.002 ± 0.001 mM versus 0.11 ± 0.01 mM at pHout 7.4, respectively, *p* < 0.01, *n* ≥ 3).

### 3.5 Further testing of Y56F-rPepT1

#### 3.5.1 Measuring Y56F-rPepT1 binding affinity for the Phe-Tyr and Phe-Tyr-NH_2_ dipeptides

**Fig. 4:**
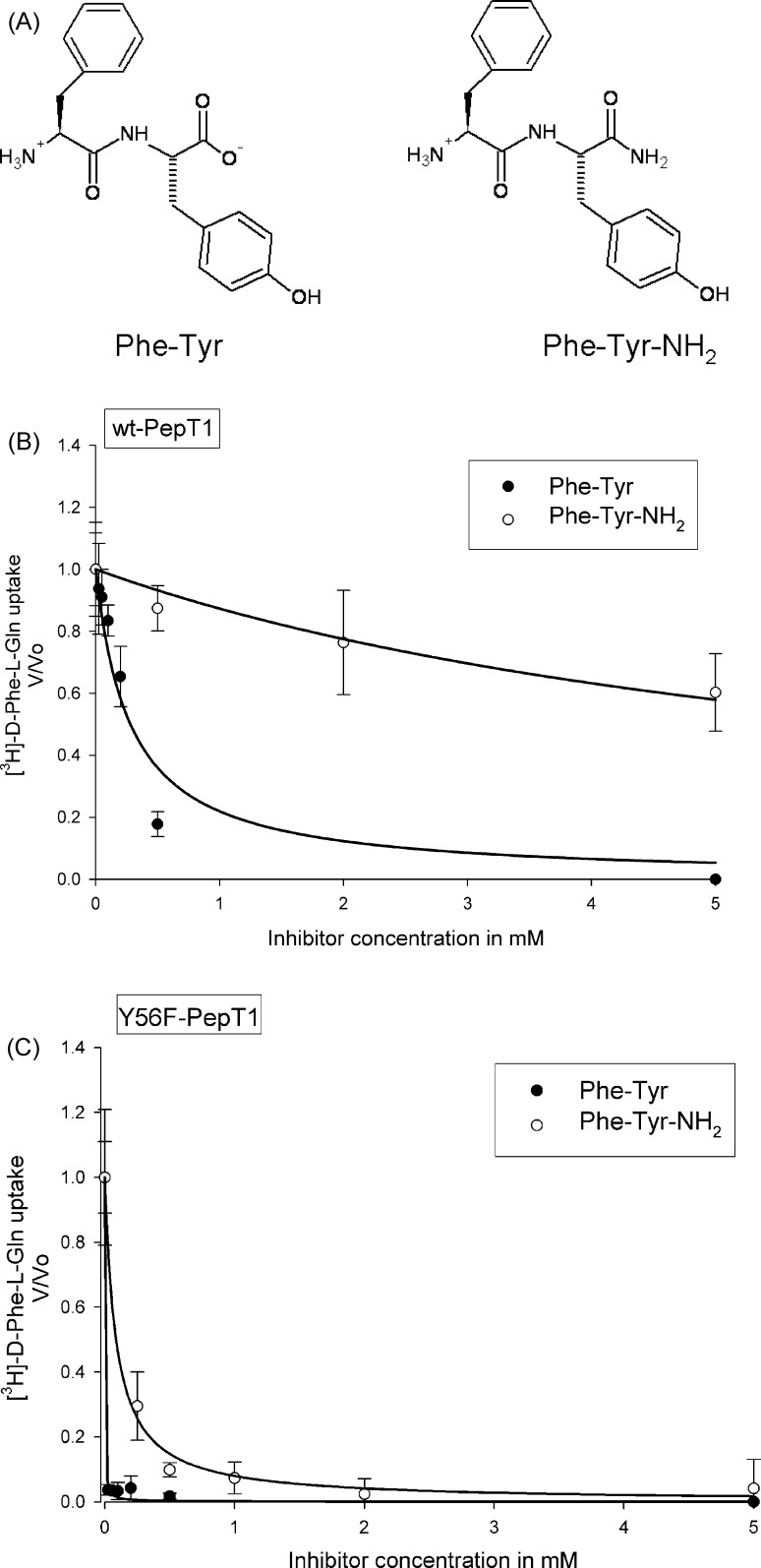
(A) Chemical structures of Phe-Tyr-NH_2_ and its parent compound Phe-Tyr. (B and C) *Ki* determination of carboxyl-terminal amidated Phe-Tyr-NH_2_ and the parent compound Phe-Tyr by wild-type-PepT1 (B) and Y56F-rPepT1 mutant (C) at pHout 5.5. Uptake of 0.4 μM [^3^H]-d-Phe-l-Gln into oocytes was measured in the presence of increasing concentrations of Phe-Tyr or Phe-Tyr-NH_2_ at pHout 5.5. Lines represent best fit of the data to Michaelis–Menten kinetics for binding to a single site (Deves and Boyd, 1989). Each data point is the mean ± SEM of 5 oocytes, and the graphs are representative of *n* = 4 oocyte preparations.

In order to further characterize the role of tyrosine56, the carboxyl-terminal amidated Phe-Tyr-NH_2_ and the parent compound Phe-Tyr (Fig. 4A) were compared for efficacy in inhibiting the uptake of [^3^H]-d-Phe-l-Gln. The increased affinity of the Y56F-rPepT1 transporter is still evident when both the parent dipeptide Phe-Tyr and the blocked C-terminal peptide Phe-Tyr-NH_2_ are used as inhibitors (*Ki* = 0.86 ± 0.26 μM and *Ki* = 72.9 ± 20.5 μM, respectively, Fig. 4B) compared to the wild-type Ki (*Ki* = 200 ± 30 μM and *Ki* = 6600 ± 1620 μM, respectively, Fig. 4C; *p* < 0.05, *n* = 4 separate experiments). The fold change in affinity was significantly smaller for Phe-Tyr-NH_2_ (fold change for *Ki* of Phe-Tyr versus Phe-Tyr-NH_2_ for wild-type rPepT1 was 336.4 ± 100.6 compared to that of Y56F-rPepT1 at 64.4 ± 26.0, *p* < 0.05, Student's *t*-test, *n* = 4).

#### 3.5.2 Uptake of [^3^H]-d-Phe-l-Gln by the single T58Y and the double Y56F/T58Y-rPepT1 mutant

**Fig. 5:**
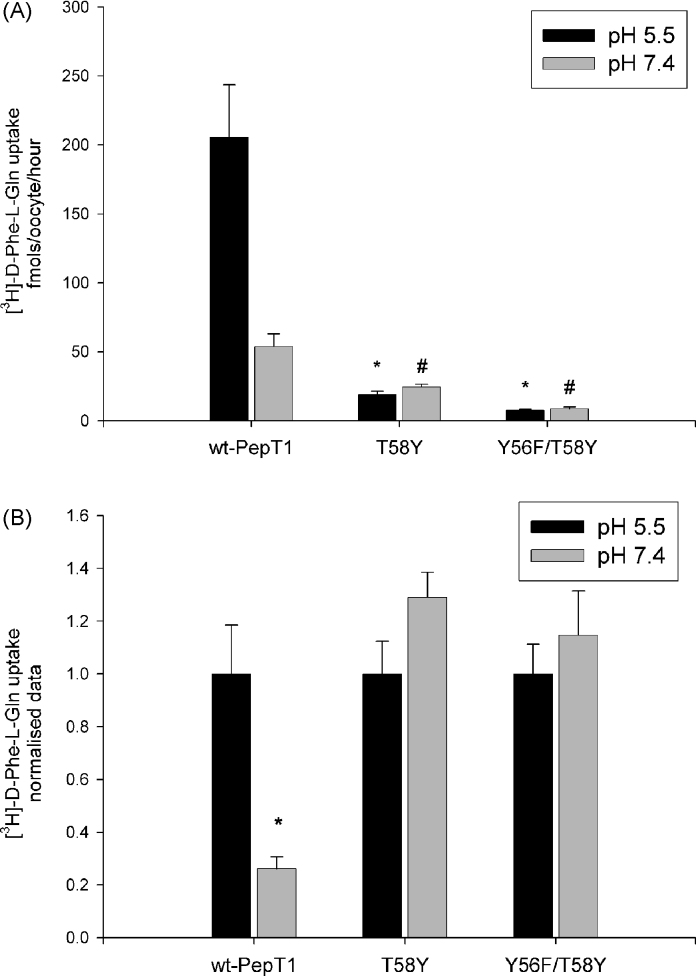
(A) Uptake of [^3^H]-d-Phe-l-Gln in *Xenopus* oocytes expressing wild-type rPepT1 (wt-PepT1), the single T58F or the double Y56F/T58Y-rPepT1 mutants. *p < 0.01 versus wild-type uptake at pHout 5.5 (black bars) and ^#^*p* < 0.01 versus wild-type uptake at pHout 7.4 (grey bars), Student's *t*-test, *n* = 3. (B) pH dependence of [^3^H]-d-Phe-l-Gln uptake normalised to the uptake of pHout 5.5 for each construct. pH dependence of uptake is seen only in the wild-type rPepT1 (*p < 0.01, Student's *t*-test, *n* = 3).

In order to further elucidate the role of tyrosine56, the double Y56F/T58Y-rPepT1 mutant was constructed and [^3^H]-d-Phe-l-Gln uptake was tested (Fig. 5A). Uptake by the single T58Y-rPepT1 mutant was used as a control. T58Y-rPepT1 transport function is significantly reduced by >70% compared to the wild-type at both pHout 5.5 and 7.4, while uptake by the double Y56F/T58Y-rPepT1 mutant is reduced by >90% compared to the wild-type transporter at both pHout 5.5 and 7.4 (*p* < 0.01, Student's *t*-test, *n* = 3). As can be seen more clearly in Fig. 5B, the pH dependence of transport was lost in both the T58Y- and Y56F/T58Y-rPepT1 mutations.

#### 3.5.3 Further mutations to tyrosine 91 (Y91)

As shown in Fig. 1A, the Y91F-rPepT1 mutant when expressed in *Xenopus* oocytes demonstrates a 40% decrease in the rate of [^3^H]-d-Phe-l-Gln transport per protein molecule. Interestingly, Y91 has previously been proposed to be important in the channel interaction with protons, and hence it was suggested that a mutation at this position would result in alterations in the pH dependence of the PepT1 peptide transporter (Bolger et al., 1998). Nonetheless, Y91 has never been tested by SDM. The importance of the residue Y91 was investigated by further mutating it to a glutamine and an arginine residue. Glutamine was postulated to be a good replacement because of its polar –(CH_2_)_2_–CONH_2_ side chain. Arginine, with a –C–(NH_2_)_2_^+^ side chain, is functionally less similar to tyrosine, but its molecular length is roughly equal to that of a tyrosine. The mutated proteins were expressed in *Xenopus* oocytes and their mode of transport was studied.

#### 3.5.4 Rate of uptake and pH dependence of transport for the Y91 mutants

**Fig. 6:**
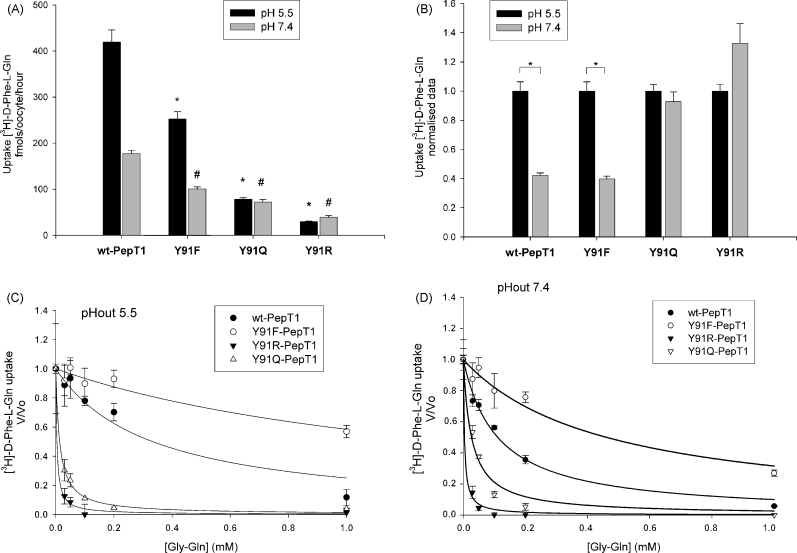
(A) Uptake of [^3^H]-d-Phe-l-Gln in *Xenopus* oocytes expressing wild-type rPepT1 (wt-PepT1), Y91F-, Y91Q- or Y91R-PepT1. *p < 0.05 versus wild-type uptake at pHout 5.5 (black bars) and ^#^*p* < 0.05 versus wild-type uptake at pHout 7.4 (grey bars), Student's *t*-test, *n* = 3). (B) pH dependence of [^3^H]-d-Phe-l-Gln uptake normalised to the uptake of pHout 5.5 for each construct. pH dependence of uptake is maintained only in the Y91F-rPepT1 mutant (*p < 0.01, Student's *t*-test, *n* = 3). (C and D) *Ki* determination of Gly-l-Gln against 0.4 μM [^3^H]-d-Phe-l-Gln uptake by wild-type rPepT1 (wt-PepT1) and the Y91F-, Y91Q- and Y91R-rPepT1 mutants at pHout 5.5 (C) and 7.4 (D). Lines represent best fit of the data to Michaelis–Menten kinetics for binding to a single site (Deves and Boyd, 1989). Each data point is the mean ± SEM of 5 oocytes, and the graphs are representative of *n* ≥ 2 oocyte preparations.

As illustrated in Fig. 6A, the rate of mediated uptake *via* all three Y91 mutants was slower than the wild-type rate, with Y91Q and Y91R being slower than Y91F-rPepT1. Interestingly, only uptake by the Y91F mutant was pH dependent whereas the uptake rate by Y91Q and Y91R mutants appeared to remain independent of the extracellular pH (Fig. 6B).

#### 3.5.5 Binding affinity determination for the Y91 mutants

Inhibition studies were carried out as previously described in order to examine whether mutating tyrosine91 alters the binding affinity of the transporter. At pHout 5.5 (Fig. 6C) the Y91F mutation caused a decrease in the affinity by which Gly-l-Gln binds to Y91F-rPepT1 as compared to the wild-type protein (*Ki* 1.40 ± 0.31 mM and 0.33 ± 0.11 mM, respectively, *p* < 0.05, *n* = 2). Moreover both the Y91Q and Y91R mutations had an effect on the binding properties of rPepT1 with an increased *Ki* of 0.013 ± 0.001 mM and 0.004 ± 0.001 mM, respectively (*p* < 0.05 compared to wild-type, *n* = 2). At pHout 7.4 (Fig. 6D) a similar pattern is observed, with Y91F causing a decrease in affinity as compared to the wild-type (0.46 ± 0.09 mM versus 0.11 ± 0.01 mM, respectively, *p* < 0.05, *n* = 2) and the Y91Q and Y91R causing an increase in affinity compared to the wild-type (0.030 ± 0.005 mM and 0.003 ± 0.001 mM, respectively, *p* < 0.05, *n* = 2).

#### 3.5.6 Further mutations to tyrosine 167

As shown in Fig. 1B the mutation of tyrosine167 to a phenylalanine resulted in a mutant transporter (Y167F-rPepT1) that when expressed on the *Xenopus* oocyte membrane demonstrated a dramatic decrease in the rate of transport down to <10% versus the wild-type. To further investigate the role of tyrosine167, site-directed mutagenesis was used to generate the Y167Q and Y167R mutants additionally to the Y167F one.

**Fig. 7:**
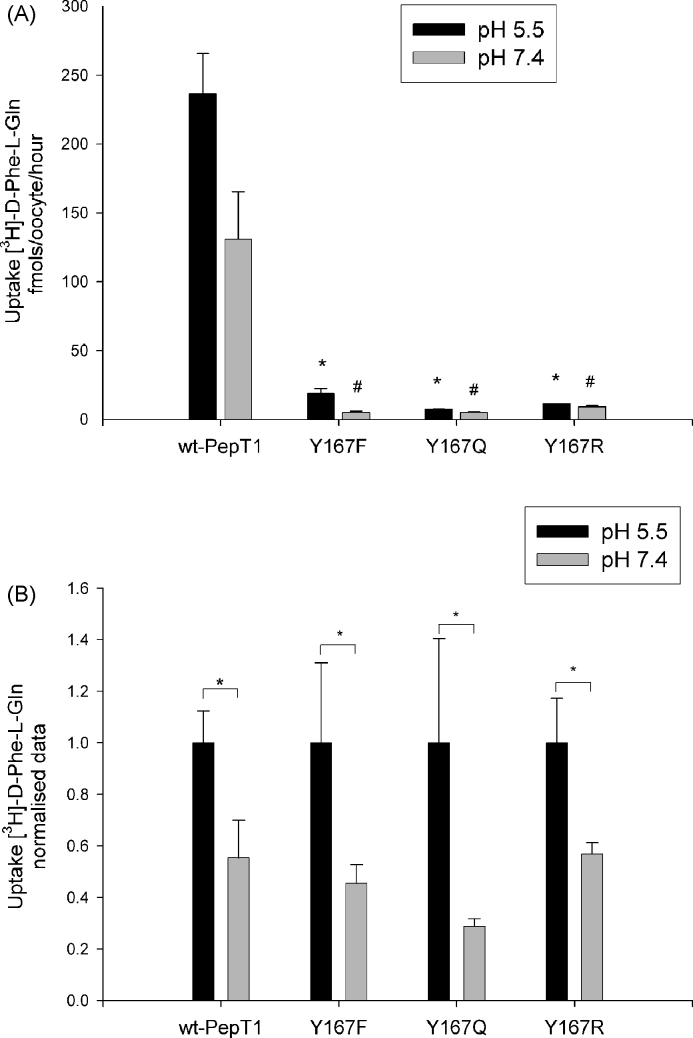
(A) Uptake of [^3^H]-d-Phe-l-Gln in *Xenopus* oocytes expressing wild-type rPepT1 (wt-PepT1), Y167F-, Y167Q- or Y167R-rPepT1 mutants at pHout 5.5 and 7.4. Uptake is reduced by >90% as compared to the wild-type at both pHout 5.5 (black bars, *p < 0.001, Student's *t*-test, *n* > 3) and pHout 7.4 (grey bars, ^#^*p* < 0.001, Student's *t*-test, *n* > 3). (B) pH dependence of [^3^H]-d-Phe-l-Gln uptake normalised to the uptake of pHout 5.5 for each construct. pH dependence of uptake is maintained in all Y167 mutants (*p < 0.05, Student's *t*-test, *n* > 3).

As shown in Fig. 7A, [^3^H]-d-Phe-l-Gln mediated uptake *via* all three Y167-rPepT1 mutants was drastically lower as compared to the wild-type transporter. Even though the signal is very small, it is still apparent that all tyrosine167 mutants seem to follow the pH dependence of the wild-type rPepT1, showing a systematic decrease in the rate of transport when the pHout is increased (from 5.5 to 7.4, Fig. 7B).

## 4 Discussion

In this study, rabbit PepT1 transmembrane tyrosines were systematically individually mutated to phenylalanine residues, the latter being the most conservative mutation that can be introduced with only the loss of an hydroxyl group. The site-directed mutants were expressed in *Xenopus* oocytes and all mutants were trafficked to the oocyte membrane, with measurement of surface expression allowing quantification of transporter function. Findings presented here show that when Y64, Y287, Y587 and Y645 were individually mutated to phenylalanine, the resulting mutant proteins retained their normal transport characteristics. The other five out of the nine tyrosines tested were identified to play a functional role in PepT1. Mutations of Y56F and Y167F caused a dramatic decrease in transport function (>90% reduction versus wild-type) whereas those of Y12F, Y91F and Y345F demonstrated a less profound, yet significant, effect (Fig. 1B). Intriguingly, none of the tyrosine to phenylalanine substitutions affected the pH dependence of the peptide transport rate, implying that the phenolic chain of the tyrosines is not essential for proton binding or translocation during the PepT1 transport cycle. This result showed that none of the tyrosine residues in the TMDs of rPepT1 was solely responsible for the proton translocation pathway. A finding such as this is not without precedent: transmembrane domain tyrosines have also been implicated in sodium binding site of the organic cation transporter OCTN2, yet it has been previously reported that substitution of all transmembrane tyrosines with phenylalanine residues in the OCTN2 transporter did not affect the sensitivity of carnitine transport to sodium stimulation (Seth et al., 1999).

### 4.1 Tyrosine56 (Y56)

The Y56F-rPepT1 transporter, when expressed in *Xenopus* oocytes, gave a protein that was normally expressed on the oocyte plasma membrane but with a rate of uptake that was greatly diminished (<10% compared to the wild-type), but nevertheless still stimulated by extracellular acidification. In order to test whether the Y56F mutation alters the affinity of the transporter for its substrate, inhibition assays were performed using the neutral dipeptide Gly-l-Gln. A striking result was that the Y56F-rPepT1 mutant had an affinity constant (*Ki*) of around 100 times lower than that of the wild-type. The finding that the affinity of Y56F-rPepT1 is so dramatically higher than the wild-type rPepT1 suggests this tyrosine either forms part of, or exerts a strong influence on, the substrate binding site of rPepT1.

Our findings confirmed those of Chen et al. (2000), who showed that transport of a neutral dipeptide (Gly-l-Leu) was reduced approximately 85% for the Y56F mutant, and lost completely with the Y56A mutation. Additionally, we have shown that the reduction in transport by Y56F was not due simply to a drop in the level of membrane expression of the mutant protein. However, in contrast to the findings of Chen et al. we recorded a large increase in affinity for neutral dipeptides (Gly-l-Gln, l-Phe-l-Tyr), whereas their earlier study demonstrated a slightly reduced affinity for their neutral dipeptide on Y56F-rPepT1 compared to the wild-type. The reason for this difference is not immediately obvious, although different methodology was used, with Chen et al. using electrophysiology (two-electrode voltage clamp), whereas in this study initial rates at tracer concentrations of radio-labelled dipeptide substrate (0.4 μM) were measured. The estimation of affinity by Chen et al. is from an indirect measure of dipeptide binding, that is the current (proton movement) that is associated with dipeptide transport, and as such should be regarded as an apparent affinity. In making their estimation of apparent affinity, Chen et al. will have had to assume that the coupling of peptides to protons remained constant; however, recently we have shown that mutations in arginine282 (R282) of rPepT1 can change the stoichiometry (Pieri et al., 2008), although in contrast to most of the R282 mutants, in the present study the Y56F mutant remained proton-coupled. In addition, an uncoupling of charge movement carried by protons would presumably result in a larger inward current (with pHout 5.5), which is difficult to reconcile with the results reported by Chen et al, especially that of a greatly reduced *I*_max_. However, as the affinities reported here are from direct measurement of dipeptide transport, they should be unaffected by such postulated changes in charge coupling.

It has previously been proposed that a conserved histidine (H57) in TMD2 is protonated and binds the carboxy terminus of the substrate (Bailey et al., 2000; Pieri et al., 2008). Y56 is the adjacent residue to histidine57 (H57) and it has been proposed that it plays a role in stabilising the positive charge on the protonated histidine, together with Y64 (Chen et al., 2000). In contrast, we propose that Y56 acts by projecting its side chain towards H57 where the phenolic group stabilizes the histidine in its unprotonated (rather than protonated) form. The introduction of the Y56F mutation retains the binding site structurally but the loss of the phenol group causes the instability of the unprotonated H57. This results in a bigger energy gain when peptides (plus proton) bind to PepT1, therefore binding is tighter (the histidine being protonated first ready to receive the dipeptide carboxyl terminus (Pieri et al., 2008)) giving the 100-fold increase in the affinity as shown in Figs. 3 and 6. However, the proton co-transport indicates that the proton must be released when the substrate is transported. The lack of a tyrosine residue to stabilize the free histidine could cause the slower release of the proton. Hence, transport rates are reduced drastically (down to <10% of the wild-type as shown Fig. 1C). An additional explanation could be that the histidine is more stable when protonated, hence it is slow to release the carboxyl group of the dipeptide. This is consistent with the reduction in the rate of transport seen with Y56F-rPepT1, with the release of the substrate from the binding site after translocation becoming the rate limiting step in the transport cycle.

To further investigate whether the Y56F mutation was affecting H57 in a role of binding the carboxy terminus of the substrate, the affinity of Phe-Tyr was compared to that of a known PepT1 substrate lacking a free carboxyl terminus, Phe-Tyr-NH_2_ (Meredith and Boyd, 2000) for both the wild-type and the Y56F PepT1. If the Y56F-rPepT1 mutant affected the binding of the substrate carboxyl terminus, then the relative effect of the mutation should be greater for the affinity of Phe-Tyr on wild-type versus mutant protein than for Phe-Tyr-NH_2_. The finding that the fold change in affinity (*Ki*) for wild-type rPepT1 versus Y56F-rPepT1 was significantly larger for Phe-Tyr than for Phe-Tyr-NH_2_ therefore supported the hypotheses that H57 is involved in binding the carboxyl terminus of the substrate and that Y56 exerts an effect on H57.

### 4.2 Threonine58 (T58)

The rationale behind the mutation of the T58Y mutant was the finding that one explanation for the effects of the Y56F mutation was a change in the local environment of H57; hence we made both a T58Y and the double Y56F/T58Y mutants. The addition of the T58Y mutant to the Y56F mutant (to give Y56F/T58Y-rPepT1) did not restore function, and the single T58Y mutant had very little activity itself. Therefore the presence of a tyrosine simply adjacent to H57 was not sufficient to give wild-type function. Intriguingly, both the single and double T58 mutants were not pH dependent, suggesting that the T58Y mutation may adversely affect the environment of H57 required for it to play its proposed role in proton coupling.

### 4.3 Tyrosine64 (Y64)

Based on the results presented here, the Y64F-rPepT1 mutant is normally expressed on the *Xenopus* oocyte membrane and the level of uptake is the same as that of the wild-type. These results are also in contrast with Chen et al. (2000) who showed that no current was measurable with a neutral dipeptide substrate when Y64F-rPepT1 was expressed in *Xenopus* oocytes. Again, the reason for this discrepancy is not clear.

### 4.4 Tyrosine91 (Y91)

**Fig. 8:**
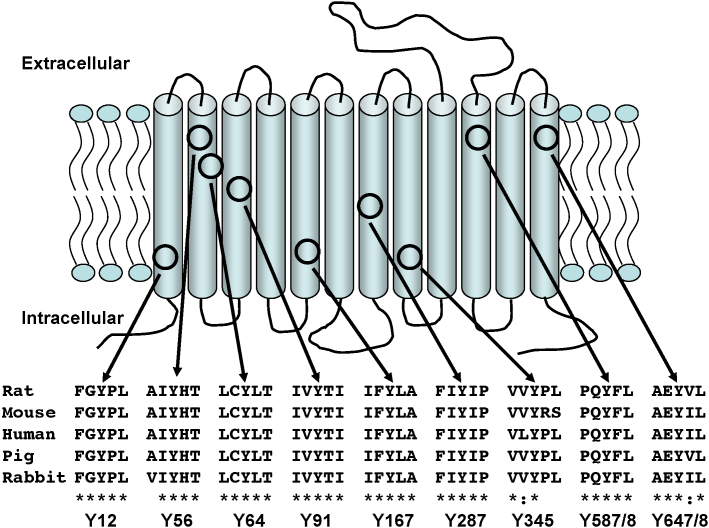
Cartoon representation of rPepT1 to show the putative position of the transmembrane tyrosines (adapted from Fei et al., 1994).

In a computer model of hPepT1 (Bolger et al., 1998), Y91 was implicated in the interaction with protons, and therefore it was predicted (but not confirmed experimentally) that a mutation at this position would result in alterations in the pH dependence of the hPepT1 peptide transporter. A recent study has shown that Y91C-hPepT1 has a transport capacity of around 25% of the wild-type, but no study on pH dependence was performed (Links et al., 2007). Results presented here show that although mutation of Y91 to a phenylalanine in rPepT1 causes a reduction in transport, transport is still stimulated by a proton gradient. No mechanism for how Y91 might be interact with protons was proposed by Bolger et al., yet it is apparent from the Y91F-rPepT1 data reported here that the phenolic group of the tyrosine is not important in this respect. In order to further investigate this residue, mutations of Y91 into an arginine (Y91R) or a glutamine (Y91Q) were made, which caused an even greater reduction in the peptide uptake despite normal trafficking to the membrane. Intriguingly, although the transport rates were low, uptake by Y91Q and Y91R was no longer proton-stimulated, supporting Bolger et al.’s original suggestion of this residue being somehow involved in the interaction of protons with PepT1. The putative position of Y91 in TMD3 would place it potentially adjacent to H57 in TMD2, tempting the hypothesis that the hydrophobic nature of residue 91 is essential to maintain the environment of H57 necessary for proton coupling (Fig. 8). In support of this, the only other mutation tested that affected proton coupling was when threonine58, i.e. the physically adjacent residue to H57, was mutated to a tyrosine.

Inhibition studies performed to determine the binding affinity revealed that the Y91F mutation showed decreased substrate binding affinity at both pHout 5.5 and 7.4. The Y91Q and Y91R mutations displayed the opposite effect, i.e. an increased affinity at both pHs. On the Kyte and Doolittle (1982) scale of hydrophobicity, phenylalanine (2.8) > tyrosine (−1.3) > glutamine (−3.5) > arginine (−4.5), and this was the rank order of affinity for the rPepT1 Y91 constructs. Therefore one possible explanation for these results is that the substrate interacts with the residue in position 91 (or one that is strongly influenced by it), hence the binding strength is determined by the hydrophobicity of the amino acid in this position. The more hydrophilic the residue, the better the substrate binds. However, the possibility that it is simply the structural differences between phenylalanine, tyrosine, arginine and glutamine that cause the effect in transport cannot be discounted.

### 4.5 Tyrosine167 (Y167)

Previously, it has been reported that mutations of Y167 in TMD5 of hPepT1 resulted in no measurable uptake of radio-labelled Gly-Sar when expressed in transfected HEK293 cells (Yeung et al., 1998). In contrast, here we show that there was measurable uptake of d-Phe-l-Gln into *Xenopus* oocytes after replacement of Y167 with phenylalanine (Y167F-), alanine (Y167A-), or glutamine (Y167Q-rPepT1), albeit at very low levels despite relatively normal surface expression of these proteins. This might be due to the increased sensitivity of the *Xenopus laevis* expression system compared to transfected HEK293 cells.

The failure of any residue to maintain wild-type transport levels when substituting Y167 shows the important role of a phenolic side chain on this residue of PepT1. These results are in agreement with the absolute conservation of Y167, which is located in the PTR motif in all mammalian H^+^/peptide cotransporters across species and homologues (reviewed by Daniel et al., 2006) and cysteine-scanning mutagenesis results showing that TMD5 forms part of the aqueous substrate translocation pathway (Kulkarni et al., 2003a). Despite the greatly reduced transport rate, the retained pH dependency of transport by all the Y167 mutants suggests that Y167 is not directly involved in the proton translocation pathway but plays a direct role (or exerts an influence) on the substrate binding and/or translocation. Due to the very small signal, it was not possible to obtain robust kinetic data, but preliminary results reveal an apparent 10-fold increase in the affinity (data not shown). Even though these data should be interpreted with caution, they do support to the hypothesis that the role of Y167 is in substrate rather than proton binding.

### 4.6 Tyrosine287 (Y287)

It has previously been reported that when Y287 is mutated to a cysteine in hPepT1, the resulting mutant protein fails to be expressed on the membrane of transfected HEK293 cells (Kulkarni et al., 2003b). In this study, Y287F-rPepT1 was expressed and functioned normally. Taken together, these results suggest that the benzoic ring is functionally and/or spatially important for wild-type-like trafficking and/or function, whereas the phenolic group appears to be dispensable.

### 4.7 Tyrosine345 (Y345)

TMDs 7, 8 and 9 of PepT1 have been previously proposed to be involved in the putative substrate binding site in PepT1/PepT2 chimeras (Fei et al., 1998) and computer modelling studies (Bolger et al., 1998). Interestingly, Y345 would be located directly below Aspartate341 (D341) in TMD8 (assuming a periodicity of 4 amino acids in the α-helix), the latter being involved in a charge pair interaction with R282 (Pieri et al., 2008). It is possible that the role of the hydroxyl group of Y345 is to stabilize the free negative charge on D341 when the salt bridge with R282 is broken during the translocation process (as proposed by Pieri et al., 2008).

### 4.8 Tyrosine587 (Y587)

In the human PepT1 protein, Y588 (analogous to Y587 in the rabbit PepT1 transporter) has previously been proposed by computer modelling to be amongst the functionally important residues (Bolger et al., 1998; Pedretti et al., 2008), but had not been tested. The suggested importance is not supported by the results presented here, since the Y587F-rPepT1 mutant was identical to the wild-type protein in terms of expression, function and pH dependence of transport.

### 4.9 Other potential transmembrane tyrosines—Y30, Y31 and Y40

Recently, homology models of PepT1 have been developed (Meredith and Price, 2006; Pedretti et al., 2008) based on the LacY permease structure (Abramson et al., 2003). These models suggest that TMD1 may not be as predicted by Fei et al. (1994) (residues 7–25) but rather more proximal and longer (residues 13–42) due to being tilted in the membrane (Meredith and Price, 2006). If the homology model were to represent the *in vivo* structure of the PepT1 protein, then Y30, Y31 and Y40 would also be transmembrane domain tyrosines. All three of these residues have also been mutated to phenylalanine: Y30F/Y31F was made as a double mutant, and was expressed but had no transport activity in oocytes, whereas Y40 was not expressed (data not shown).  

In conclusion, this systematic investigation of conserved tyrosines located in the TMDs of rPepT1 failed to reveal any residue for which the phenolic characteristics were essential for proton coupling of peptide transport, contrary to predictions in the literature. However, when Y91 was mutated to hydrophilic residues, namely Y91Q and Y91R, the proton coupling was lost, as it was with the T58Y mutation. The finding of a similar effect for both Y91Q/R mutants as for Y58F provides support for the speculation that Y91 may play a role in pH stimulation of transport by exerting an influence on H57.

## Figures and Tables

**Table 1:** Primer sequences used to generate the rabbit PepT1 mutations described. The reverse primer was the reverse compliment of the respective forward primer.

Mutant	Primer	Primer sequence (5′ → 3′)
Y12F-rPepT1	Forward	C TGA GCT GCT TCG GCT **TTC** CCC TGA GCA TCT TC
Y30F/Y31F-rPepT1	Forward	GCGAAAGGTTCTCC**TTCTTT**CCCATGAGAGCACTC
Y40F-rPepT1	Forward	AGCACTCCTGATTCTG**TTC**TTCAGAAACTTCATCG
Y56F-rPepT1	Forward	G TCC ACG GTC ATC **TTC** CAC ACG TTC GTC G
Y64F-rPepT1	Forward	TTC GTC GCG CTG TGC **TTC** CTC ACG CCC ATT CTC
Y91F-rPepT1	Forward	G TGG CTG TCT ATC GTC **TTC** ACC ATC GGA CAA GCA G
Y91Q-rPepT1	Forward	G TGG CTG TCT ATC GTC **CAA** CCA TCG GAC AAG CAG
Y91R-rPepT1	Forward	C GTG TGG CTG TCT ATC GTC **AGA** ACC ATC GGA CAA GCA GGA C
Y167F-rPepT1	Forward	A ACC GGT TTT TTT CCA TCT **TTC** TTG GCC ATT AAC GCT GGG AGT
Y167Q-rPepT1	Forward	A ACC GGT TTT TTT CCA TCT **CAA** TTG GCC ATT AAC GCT GGG AGT
Y167R-rPepT1	Forward	C GGT TTT TTT CCA TCT **CGC** TTG GCC ATT AAC GCT GGG
Y287F-rPepT1	Forward	G GTG CTG TTC CTG **TTC** ATC CCA CTC CCC A
Y345F-rPepT1	Forward	A TGG ACG CCG TGG TGT **TTC** CTC TGA TTG CAA AG
Y587F-rPepT1	Forward	T TGG CAA ATC CCA CAG **TTC** TTC CTC ATC ACC TCT G
Y645F-rPepT1	Forward	AAG CAG TGG GCC GAG **TTC** ATC CTC TTT GCC GCC
T58F-rPepT1	Forward	ACG GTC ATC TTC CAC **TTT** TTC GTC GAT GTG
Y56F/T58F-rPepT1	Forward	G TCC ACG GTC ATC **TTC** CAC ACG TTC GTC G
